# Structural and functional impact of the p.R163C mutation in the conserved palindromic motif within the C-terminal domain of human αB-crystallin

**DOI:** 10.1371/journal.pone.0326025

**Published:** 2025-07-14

**Authors:** Aref Baharvand, Zamara Mariam, Mohammad Bagher Shahsavani, Leila Rezaei Somee, Issa Zarei, Massoud Amanlou, Giuseppe Deganutti, Ali Akbar Saboury, Ali Akbar Moosavi-Movahedi, Reza Yousefi

**Affiliations:** 1 Protein Chemistry Laboratory (PCL), Institute of Biochemistry and Biophysics (IBB), University of Tehran, Tehran, Iran; 2 Centre for Sport, Exercise and Life Sciences (CSELS), Faculty of Health and Life Sciences, Coventry University, Coventry, United Kingdom; 3 Department of Biology, Shiraz University, Shiraz, Iran; 4 Department of Medicinal Chemistry, Faculty of Pharmacy, Tehran University of Medical Sciences, Tehran, Iran; 5 Institute of Biochemistry and Biophysics (IBB), University of Tehran, Tehran, Iran; Panjab University Chandigarh, INDIA

## Abstract

Human αB-crystallin is a small heat shock protein that functions as a chaperone and anti-apoptotic protein to maintain cellular protein integrity. A specific mutation (p.R163C) in the C-terminal domain has been linked to dilated cardiomyopathy (DCM). However, the impact of this mutation on the protein’s structure, activity, stability, and amyloidogenic properties remains unclear. Here, we introduced the mutation, expressed and purified the protein, and used spectroscopic and microscopic techniques to conduct a comprehensive investigation of the mutant protein. The p.R163C mutation in αB-crystallin induces subtle changes in its secondary and tertiary structures, resulting in a slight increase in the distance and angle between monomer units within the dimer. The mutation causes the protein to form larger oligomers with increased chaperone activity, which may protect against cell death but could also lead to excessive client protein sequestration or coaggregation, potentially causing cytotoxicity. Accompanied by these alterations, the chemical and thermal stability of the mutant protein decrease, the resistance of the protein to enzymatic digestion increases, and finally, the propensity of the p.R163C mutated protein to form amyloid fibrils elevates. The substitution of the conserved arginine at position 163 with cysteine likely impacts the ability of the mutated protein to interact with cardiac muscle proteins. Collectively, these structural and functional modifications in the mutated protein may perturb cellular homeostasis and contribute to the onset of DCM.

## 1. Introduction

Small heat shock proteins (sHsps) are the most ubiquitous and diverse group of molecular chaperones that sequester misfolded proteins in an ATP-independent manner to hinder the formation of insoluble, cytotoxic protein aggregates [1,2]. Among the ten members of the human sHsps family, αB-crystallin stands out due to its broad expression and large interactome of over 115 target proteins within various tissues, particularly the heart, brain, and eye lens [3,4]. Upon cellular stress, αB-crystallin is upregulated and suppresses amorphous and amyloid protein aggregation by holding misfolded clients with distinguished structural interfaces [5,6]. These captured non-native protein conformations are later refolded via ATP-dependent Hsp70-Hsp100 chaperones, either targeted for degradation in ubiquitin-proteasome or autophagy systems [7]. Apart from chaperone activity, αB-crystallin is also known to inhibit apoptosis by interacting with key apoptotic regulators such as Bax and Bcl-X_S_, preventing mitochondrial cytochrome c release and subsequent caspase activation [8].

The primary structure of human αB-crystallin, similar to other small heat shock proteins, organizes into three distinct domains [9,10]. The α-crystallin domain (ACD, residues 66–148) is central to this structure, primarily composed of β-sheets and mediates homodimerization through the antiparallel interaction of the β6/7 strands from two ACDs [11]. Flanking this core domain are flexible N-terminal (NTD, residues 1–65) and C-terminal (CTD, residues 149–175) domains, contributing to protein oligomerization [12,13]. Previous findings revealed many functional sequences across the 175 amino acids of αB-crystallin. For instance, the CTD contains an IXI/V motif, which binds to the hydrophobic groove between the β4 and β8 strands of the ACD from a partner dimer and leads to the assembly of hexamers [14]. The dimers and hexamers serve as fundamental building blocks for generating larger oligomeric complexes [11]. Moreover, this motif is in a conserved palindromic peptide sequence 156ERTIPITRE164 that is recognized as a mini-chaperone and has a preventive role in protein aggregation [15–17]. This peptide in the CTD is also a subunit-subunit interaction site in human αB-crystallin, which generates a pattern of hydrophobic patches in its three-dimensional surface [18,19]. Arginine residues are critical in maintaining protein structure and stability. Notably, the arginine within the conserved REEK motif, shared between αA- and αB-crystallin is essential for proper oligomerization [20]. It is worth mentioning that the amino acid sequence of αB-crystallin lacks cysteine residues. Consequently, the native protein lacks both intra- and intermolecular disulfide bonds, ensuring its structural integrity remains independent of this covalent cross-linking. However, certain disease-associated mutations introduce cysteine substitutions. For example, the substitution of arginine with cysteine at position 163 (R163C) is a critical mutation linked to the development of dilated cardiomyopathy (DCM), and it serves as the primary focus of this study [21,22]. DCM is the second leading cause of heart transplantation after coronary artery disease [23]. It is characterized by the dilation of the heart chambers, impaired contractile function of the heart muscle, and reduced cardiac output. The pathogenic variants in the *CRYAB* gene, which encodes αB-crystallin, may contribute to disease progression by disrupting interactions of αB-crystallin with key cardiac proteins and altering the subcellular localization of the mutant protein under stress conditions [24–26].

**Scheme 1:**
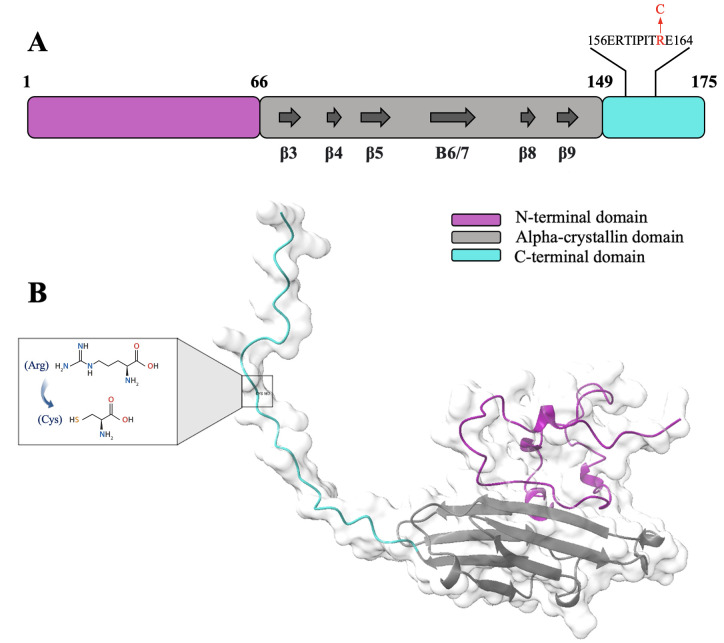
Domain organization of human αB-crystallin protein. **(A)** The human αB-crystallin protein consists of 175 amino acids organized into three key domains, each with unique structural and functional attributes. The N-terminal domain facilitates the formation of protein oligomers. The ACD, which is primarily made up of β-strands, is vital for chaperone activity and the dimerization of proteins. The C-terminal domain, containing the 156ERTIPITRE164 palindrome, which the R163C mutation located within this region, plays an important role in interactions with other proteins and contributes to chaperone function [27]. **(B)** AlphaFold2-predicted structure of monomeric p.R163C αB-crystallin and the mutation site [28].

Given the importance of Arginine 163 within the conserved palindromic sequence in the C-terminal domain, its substitution with cysteine, as illustrated in Scheme 1, in addition to its association with DCM, introduces a novel residue into αB-crystallin, potentially creating a structural and functional hotspot.

Therefore, this study aims to investigate the impact of this mutation within the protein’s flexible domain, assessing its influence on structure, stability, oligomerization, and chaperone activity.

## 2. Materials and methods

### 2.1 Materials

1-Anilino-8-naphthalene sulfonic acid (ANS), Thioflavin T (ThT), α-glucosidase (α-Gls), α-chymotrypsin, bovine pancreatic insulin, isopropyl β-D-1-thiogalactopyranoside (IPTG), dithiothreitol (DTT), diethyl aminoethyl (DEAE) cellulose and kanamycin were purchased from Sigma. β-Mercaptoethanol (β-ME), ethylenediaminetetraacetic acid (EDTA), urea, and other chemicals were obtained from Merck.

### 2.2 Site-directed mutagenesis, protein expression, and purification

The cDNA encoding wild-type human αB-crystallin was cloned into the pET-28b(+) vector. To generate site-directed mutation, the QuikChange II XL Site-Directed Mutagenesis Kit (Stratagene) was utilized with the forward primer 5′-CATTCCCATCACCTGCGAAGAGAAGCCTG-3′ and the reverse primer 5′-CAGGCTTCTCTTCGCAGGTGATGGGAATG-3′. The introduction of mutation was confirmed by DNA sequencing. The recombinant plasmid was transformed into *Escherichia coli* (*E.coli*) BL21, DE3 cells. Cultures were grown in Luria-Bertani (LB) medium containing 50 μg/mL kanamycin at 37 °C with agitation at 150 rpm. Protein expression was induced by adding 0.25 mM IPTG when cultures reached an optical density of 0.6 at 600 nm (OD600). Post-induction, cultures were incubated at 37 °C overnight to optimize protein yield and solubility. Cells were harvested by centrifugation at 8000 rpm for 15 minutes at 4 °C. Pellets were resuspended in a lysis buffer comprising 40 mM Tris-HCl (pH 8.0), 8 M urea, and 10 mM β-ME, followed by incubation at 37 °C for 30 minutes to facilitate protein extraction. Lysates were cleared by centrifugation at 14,000 rpm for 30 minutes at 4 °C and subsequently applied to a DEAE-cellulose anion exchange chromatography column equilibrated with 20 mM Tris-HCl (pH 8.0), 4 M urea, and 5 mM β-ME. Proteins were eluted using NaCl, and the eluted fractions were analyzed for purity using SDS-PAGE. Dialysis against double distilled water (ddH_2_O) was performed to remove urea, and the purified proteins were lyophilized and stored at −20 °C [29,30].

### 2.3 Mass spectrometry analyses

The molecular mass of wild-type and mutant proteins was evaluated by an electrospray ionization-quadrupole time-of-flight (ESI-QTOF) mass spectrometry using a Waters Synapt G1 instrument. Protein samples with a concentration of 0.2 mg/mL were mixed with distilled water and infused into the spectrometer at a flow rate of 30 μL/min. Mass spectra were acquired in the m/z range of 600–2000 for monomeric proteins. Deconvolution of the raw data was performed using the MassLynx MS software to determine the accurate mass of the proteins [31].

### 2.4 Circular dichroism (CD) assessment

Recombinant human αB-crystallin proteins were prepared in buffer A (50 mM phosphate buffer, pH 7.4) at 1.5 mg/mL for near-UV CD and 0.2 mg/mL for far-UV CD spectroscopy. Measurements were conducted using an Aviv 215 CD spectropolarimeter (USA) with cuvette path lengths of 1 cm and 0.1 cm, respectively. Molar ellipticity data were analyzed using the BeStSel server to determine secondary structure content (https://bestsel.elte.hu/index.php) [32,33].

### 2.5 Fourier-transform infrared (FTIR) and Raman studies

The secondary structure of αB-crystallin wild-type and mutant (p.R163C) proteins was analyzed using FTIR and Raman spectroscopy. FTIR spectra were recorded using a Tensor II spectrometer in the 1550–1742 cm^−1^ region (resolution: 4 cm^−1^, 256 scans). The Amide I region (1610–1700 cm^−1^) was analyzed using GRAMS/AI™ 9.2 software with Gaussian deconvolution, identifying peaks for β-sheet (1610–1642 cm^−1^), random coil (1643–1650 cm^−1^), α-helix (1650–1659 cm^−1^), and β-turn (1660–1699 cm^−1^). Raman spectroscopy was performed with a Lab Ram HR spectrometer using a 633 nm red laser, 240-second integration, and 17 mW power. Spectra were recorded in the 600–1800 cm^−1^ region, focusing on the Amide I range (1620–1700 cm^−1^). Peaks identified included β-sheet (1620–1648 cm^−1^ and 1665–1680 cm^−1^), α-helix (1649–1660 cm^−1^), random coil (1660–1665 cm^−1^), and β-turn (1658–1699 cm^−1^). Data analysis and deconvolution were performed using GRAMS/AI™ 9.2 software [34,35].

### 2.6 Fluorescence spectroscopy studies

Fluorescence spectroscopy was used to evaluate structural changes in αB-crystallin proteins at a concentration of 0.15 mg/mL in buffer A. Intrinsic fluorescence of tryptophan (Trp) and tyrosine (Tyr) was measured using a Varian Cary Eclipse spectrofluorometer at 27 °C, 37 °C, and 47 °C. Excitation wavelengths were set to 295 nm (Trp) and 280 nm (Tyr), with slit widths of 5/10 nm. Emission spectra (300–500 nm) and synchronous spectra (200–350 nm) were recorded using excitation intervals of 60 nm for Trp and 15 nm for Tyr [29]. Additionally, three-dimensional (3D) fluorescence spectra and contour maps were obtained by exciting the samples at 200–350 nm with 5 nm intervals and recording emissions at 300–350 nm. Scanning speed, detector voltage, and slit widths were set to 2400 nm/min, 800 V, and 5/10 nm, respectively [36]. An ANS probe was employed for hydrophobic surface analysis. Protein samples (0.15 mg/mL) were mixed with 100 µM ANS and incubated in the dark for 30 minutes. Emission spectra (400–600 nm) were recorded after excitation at 365 nm by the spectrofluorometer, with slit widths set to 5/10 nm [36,37].

### 2.7 Molecular dynamics (MD) simulation

MD simulations were conducted to investigate the dimeric structure of the p.R163C αB-crystallin mutant using the CHARMM36 force field. The systems preparation was carried out with high-throughput molecular dynamics (HTMD) [38], a Python-based tool for large-scale molecular modeling, and tool command language (TCL) scripting for workflow control. Hydrogen atoms were added at a simulated pH of 7.4 using protein data bank identifier (PDB ID): 2PQR and PropKa, which assigned atomic charges, optimized protonation states, and predicted pKa values [39,40]. The systems were neutralized and solvated in water using visual molecular dynamics (VMD) autoionize and solvate tools. MD simulations were performed with accelerated molecular dynamics (ACEMD) [41], including a 4 ns equilibration phase during which restraints were applied to the protein Cα atoms and gradually released, followed by 3000 ns of production, leveraging GPU-optimized computing for efficiency.

### 2.8 Multiple walkers supervised molecular dynamics (mwSuMD) simulations

The conformational transitions between the two subunits of αB-crystallin were explored using an unbiased adaptive sampling method called mwSuMD [42]. This method accelerates the simulation of transition states, including binding or unbinding events. For this study, the distance between the centroid of Cys163 of both αB-crystallin subunits of the mutant was supervised. The single metric score (SMscore) was employed to determine which walker progressed in the simulation. After each batch, the walker with the highest SMscore was selected for extension, and a new batch of walkers (200 ps each) was seeded from it.

### 2.9 Protein oligomerization assessments

To investigate the impact of the p.R163C mutation on the oligomeric size and morphology of human αB-crystallin, we utilized both dynamic light scattering (DLS) and atomic force microscopy (AFM). For the DLS analysis, wild-type and mutant protein samples were prepared at a concentration of 1 mg/mL in buffer A and measured with an SZ-100 instrument using a 532 nm laser at a scattering angle of 173°. For AFM imaging, 3 μL of each protein sample at a concentration of 0.025 mg/mL was applied to freshly cleaved mica discs and allowed to air-dry at room temperature. Images were captured in non-contact mode with a silicon nitride AFM cantilever. Image processing and analysis of particle size distribution were conducted using Imager (version 1.01) and Gwyddion (version 2.62) software.

### 2.10 Protein stability assessments

Protein stability is defined as the standard Gibbs free energy difference (*ΔG*^*°*^) between the native and unfolded states. In this study, the stability of wild-type and mutant (p.R163C) human αB-crystallin was analyzed using urea-induced denaturation. Protein samples (0.15 mg/mL in buffer A) were incubated with increasing urea concentrations (0–8 M) for 18 hours at room temperature. Fluorescence emission spectra (excitation at 295 nm) were recorded from 300 to 500 nm. A three-state denaturation model was applied, plotting fluorescence intensity ratios (*I*_*U*_*/I*_*N*_) against urea concentration. Quantitative parameters were derived using the equation:


$$F=\frac{F_{N}+\frac{F_{1}\exp\left(-\Delta G_{1}^{0}+m_{1}[Ur𝕖a]\right)}{RT}+F_{U}\exp(\Delta G_{2}^{0}+ma,[Urea])/RT}{1+\frac{\exp\left(-\Delta G_{1}^{0}+m_{1}[Ur𝕖a]\right)}{RT}+\exp(-\Delta G_{2}^{0}+ma,[Urea])/RT}$$

where F_U_, F_1,_ and F_N_ represent fluorescence intensity ratios for the unfolded, intermediate, and native states, respectively. *ΔG*^*°*^_*1*_ and *ΔG*^*°*^_*2*_ correspond to standard Gibbs free energy changes between these states, with *ΔG*^*°*^ representing the overall denaturation standard Gibbs free energy [43]. Furthermore, differential scanning calorimetry (DSC) was used to assess protein thermal stability with an N-DSC II model 6100 calorimeter. Human αB-crystallin samples (1.5 mg/mL in buffer A) were heated from 25 to 85 °C at 1 °C per minute. Data were analyzed using CpCalc 2.1 software, and thermodynamic parameters—including enthalpy (*ΔH*), heat capacity (*ΔCp*), entropy (*ΔS*), melting temperature (*T*_*m*_), and free energy change (*ΔG*) were determined. The proteolytic stability of the mutant (p.R163C) αB-crystallin against enzymatic digestion by α-chymotrypsin was also studied and compared to the wild-type. Protein samples (1 mg/mL in buffer A) were incubated with α-chymotrypsin (100:1 protein-to-enzyme ratio) at 37 °C for 0, 5, 10, 15, and 20 minutes. The reaction was terminated by heating at 100 °C for 10 minutes. Subsequently, 12.5 μg of each sample was loaded onto a 12% SDS-PAGE gel, stained, and destained to evaluate proteolytic stability [44].

### 2.11 Amyloid fibril analyses

The study investigated the tendency of αB-crystallin proteins to form amyloid fibrils under thermochemical stress conditions. Proteins (2 mg/mL) were incubated at 60 °C with 1 M guanidine hydrochloride for four days. Subsequently, samples (0.15 mg/mL) were mixed with 20 μM ThT and incubated in the dark for 5 minutes before recording fluorescence emission (450–600 nm) upon excitation at 440 nm [45]. For fluorescence microscopy, 5 μL of ThT-stained samples were placed on glass slides and imaged using an Axioskop 2 Plus microscope with a GFP filter (excitation 469 nm and emission 525 nm). The amyloid fibril morphology was further analyzed using AFM. Fibril samples were centrifuged at 14,000 rpm for 30 min at 4 °C), resuspended in buffer A, and deposited onto mica discs. After drying and rinsing, images were captured in non-contact mode using an ARA-AFM system. Particle distribution analysis was performed using Imager (v1.01) and Gwyddion (v2.62) [43].

### 2.12 Chaperone activity studies

The chaperone activity of p.R163C αB-crystallin was assessed using catalase (0.3 mg/mL), γ-crystallin (0.25 mg/mL), and insulin (0.3 mg/mL) as target proteins. Aggregation was induced at 60 °C for catalase and γ-crystallin, and at 40 °C with 20 mM DTT for insulin. Light absorption at 360 nm was recorded using a Cary 100 Bio UV-Vis spectrophotometer. Similar to the previous studies, chaperone efficiency was quantified by calculating the percentage of protection, where a lower area under the curve indicates higher protection [46]. *E. coli* expressing wild-type or mutant αB-crystallin was grown at 37 °C (control) and 50 °C (heat stress). Colony survival was compared to bacteria containing an empty vector. Recombinant expression was confirmed via SDS-PAGE following IPTG induction. Viability was assessed by calculating the ratio of colonies at 50 °C to 37 °C. α-Glucosidase (0.2 U/mL) was incubated at 46 °C in the presence or absence of 0.05 mg/mL αB-crystallin (wild-type or mutant). Enzymatic activity was monitored every 5 minutes for 30 minutes

γ-Crystallin and αB-crystallin (1 mg/mL, 1:1 ratio) were incubated at 37 °C for 10 days. Aggregation was analyzed via SDS-PAGE after centrifugation (14,000 rpm, 10 min, 4 °C) [47].

### 2.13 MTT assay

SH-SY5Y neuroblastoma cells were grown in Dulbecco’s Modified Eagle Medium/Nutrient Mixture F-12 (DMEM/F12), supplemented with fetal bovine serum (FBS) and penicillin/streptomycin antibiotics, under standard conditions of 37 °C and 5% CO_2_. The cells were plated at a density of 10,000 cells per well in 96-well plates and allowed to attach overnight. To assess the protective role of αB-crystallin against oxidative stress induced by H_2_O_2_, cells were treated with 25 and 12.5 μM of αB-crystallin in serum-free media for 2 hours before exposure to 0.8 mM H_2_O_2_. After the H_2_O_2_ treatment, cell viability was assessed using the MTT assay, which detects the reduction of the yellow tetrazolium dye MTT to purple formazan crystals by the mitochondrial dehydrogenases in viable cells. Following the 2-hour αB-crystallin pre-treatment and H_2_O_2_ exposure, the culture medium was replaced with 100 μL of serum-free media containing 0.15 mg/mL MTT. The resulting formazan was dissolved in dimethyl sulfoxide (DMSO) and its absorbance was measured at 570 nm using an ELISA reader (Bio Tek, USA) [48].

### 2.14 Statistical analysis

Statistical analysis of the data was performed using a t-test and One-way ANOVA with GraphPad Prism 9 software. Statistical significance in the results was determined using analysis of variance and p < 0.05 was reported as a significant difference.

## 3. Results

### 3.1 Mutagenesis, purification, and validation of the p.R163C human αB-crystallin

**Fig 1:**
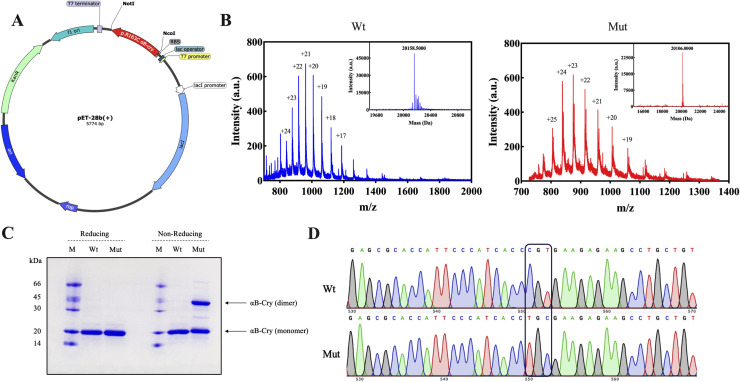
Gene mapping, mass spectrometry, and purification of the wild-type and p.R163C mutant of human αB-crystallin. **(A)** Schematic representation of the pET-28b(+) plasmid harboring the cDNA of human αB-crystallin with the p.R163C mutation, introduced via site-directed mutagenesis. **(B)** ESI-QTOF mass spectrometry analysis of purified human αB-crystallin, with spectra acquired in the m/z range of 600–2000 to confirm the molecular mass of wild-type (Wt) and mutant (Mut) forms. **(C)** SDS-PAGE analysis of the purified αB-crystallin and p.R163C mutant indicates that their monomeric molecular weight is approximately 20 kDa. Nonetheless, in the absence of a reducing agent, the p.R163C mutant forms a disulfide-linked dimer, detectable as a higher molecular weight band. **(D)** DNA sequencing chromatogram validating the p.R163C mutation (CGT to TGC) in the αB-crystallin gene, confirming successful mutagenesis at codon 163.

The p.R163C mutation in the conserved palindromic peptide sequence was introduced into a recombinant plasmid containing the αB-crystallin coding gene using site-directed mutagenesis (Fig 1A). Following the DNA sequencing to validate the implementation of the desired mutation (Fig 1B), both wild-type and mutant αB-crystallin were expressed in the BL21 (DE3) strain of *E. coli* using IPTG induction and purified via DEAE cellulose column chromatography.

The purity of proteins and the potential formation of disulfide-linked dimers were subsequently assessed with SDS-PAGE (Fig 1C). Furthermore, deconvoluted ESI-QTOF mass spectra revealed molecular weights of 20,158.5 Da for the wild-type protein and 20,106.0 Da for the mutant, which confirms the p.R163C mutation with a mass difference of 52.5 Da, corresponding to the substitution of arginine with cysteine. These findings validate the introduction of the mutation and indicate no structural damage occurred during protein expression or purification (Fig 1D).

### 3.2 Structural changes in human αB-crystallin driven by p.R163C mutation

**Fig 2:**
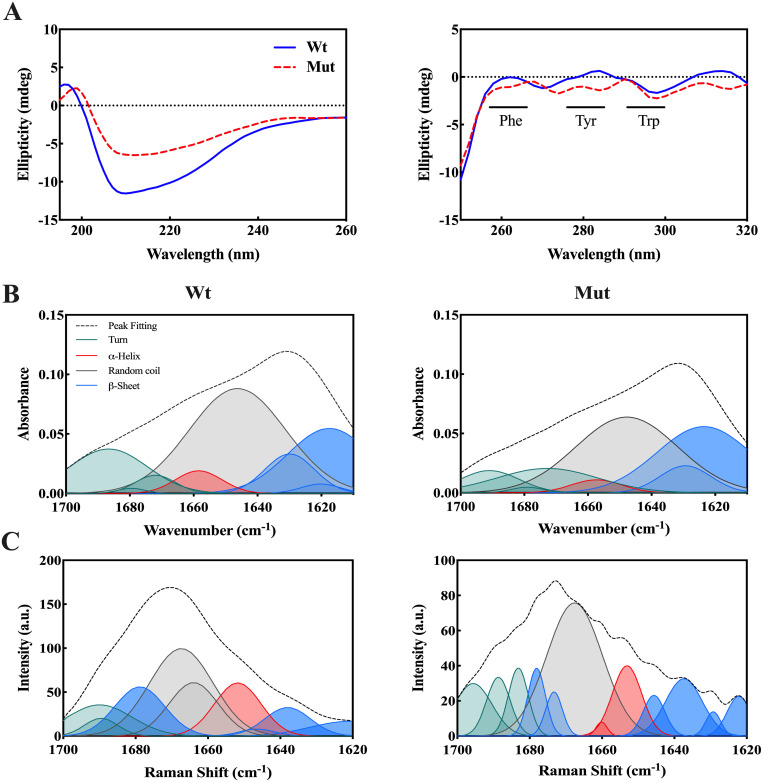
Spectroscopic characterization of wild-type and p.R163C αB-crystallin: CD, FTIR and Raman analysis. **(A)** CD spectra of recombinant human αB-crystallin. Left: Far-UV CD spectrum, showing secondary structure features. Right: Near-UV CD spectrum, reflecting tertiary structure differences. **(B)** Deconvoluted FTIR spectra of wild-type and p.R163C αB-crystallin in the Amide I region (1610–1700 cm ⁻ ¹). **(C)** Deconvoluted Raman spectra of wild-type and p.R163C αB-crystallin in the Amide I region (1620–1700 cm ⁻ ¹).

Far-UV CD analysis revealed slight differences in the secondary structural content between the wild-type and mutant proteins. The mutant protein demonstrated a modest increase in β-sheet and β-turn content, while the levels of α-helix and random coil structures showed a slight decrease relative to the wild-type protein (Table 1). Near-UV CD spectra suggested minor reductions in ellipticity for the mutant protein, particularly in regions linked to phenylalanine and Tyr residues, indicating localized changes in the environment of these aromatic residues. No significant changes were observed for Trp residues. These results suggest that the p.R163C mutation does not induce substantial alterations in the secondary or tertiary structure of αB-crystallin (Fig 2A).

**Table 1:** The percentage composition of secondary structure elements in wild-type and mutant αB-crystallin, as determined by CD, FTIR, and Raman spectroscopy.

Method	αB-crystallin	α-Helix	β-Sheet	β-Turn	Random coil
CD	Wild-type	6.8	26.0	14.0	53.2
	p.R163C	5.2	30.7	15.2	48.9
FTIR	Wild-type	4.7	32.7	17.2	45.4
	p.R163C	3.4	38.7	18.2	40.0
Raman	Wild-type	14.1	26.1	13.3	46.5
	p.R163C	11.1	30.0	21.8	37.1

To assess the secondary structural changes in human αB-crystallin caused by the p.R163C mutation, FTIR and Raman spectroscopy analyses were also conducted. Fig 2B and 2C show the FTIR and Raman spectra of the secondary structure types in the Amide I region, respectively. The quantified values of the secondary structures are also presented in Table 1, which were obtained by calculating the ratio of the area under each plot to the total plot. Based on these data, the p.R163C mutation leads to an increase in the percentage of β-sheets and β-turns, along with a decrease in the content of α-helical and random coil structures. These structural alterations are also consistent with the findings from the Far-UV CD spectrum analysis.

**Fig 3:**
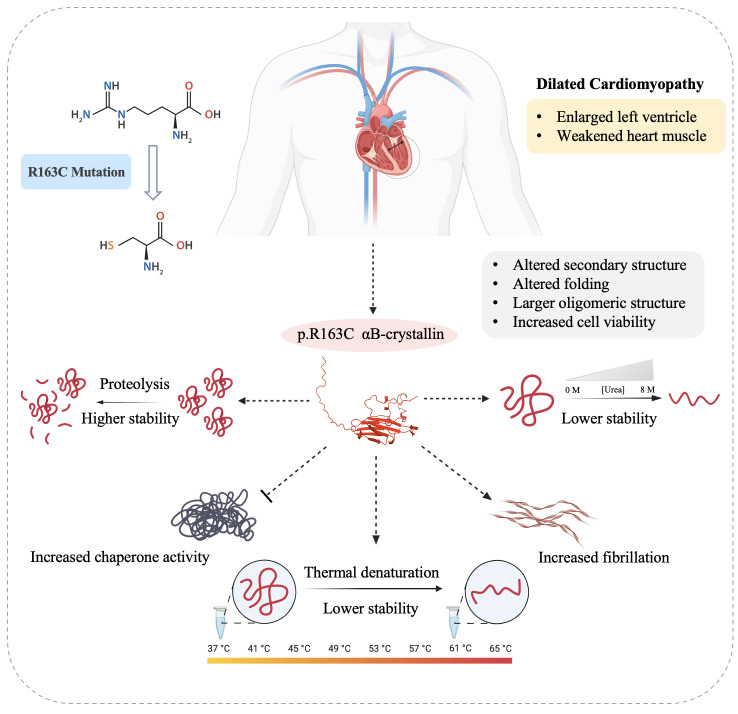
Raman spectroscopy was employed to investigate the conformational variations between wild-type and p.R163C mutant αB-crystallin proteins. Raman spectra of wild-type and p.R163C αB-crystallin were acquired, spanning the 600–1800 cm ⁻ ¹ region. This structural fingerprint region highlights the prominent β-sheet peaks at 1671 cm ⁻ ¹ (Amide I) and ~1240 cm ⁻ ¹ (Amide III) in both variants, alongside a minor α-helix peak at ~935 cm ⁻ ¹. The Trp Fermi doublet ratio (I_1360_/I_1340_) is 0.94 for the wild-type and 0.88 for the mutant, suggesting a more hydrophilic Trp environment in the p.R163C variant. The Tyr Fermi doublet ratio (I_850_/I_830_) is 0.96 for the wild-type and 0.94 for the mutant, indicating Tyr phenol side chains predominantly act as hydrogen bond donors in both proteins. These findings reveal subtle structural distinctions induced by the p.R163C mutation.

Further insights from Raman spectroscopy suggested that both wild-type and mutant proteins have distinct bands in the regions of 1671 cm^−1^ (Amide I) and near 1240 cm^−1^ (Amide III), which are characteristic of the β-structure in proteins. Moreover, both proteins have a small band near 935 cm^−1^, which can be attributed to the α-helical structure. The Trp Fermi doublet ratio (0.94 for wild-type, 0.88 for mutant) indicated a hydrophilic environment surrounding Trp residues. Meanwhile, the Tyr Fermi doublet ratio (0.96 for wild type, 0.94 for mutant) revealed that the Tyr phenol side chains primarily act as hydrogen bond donors in both variants (Fig 3).

**Fig 4:**
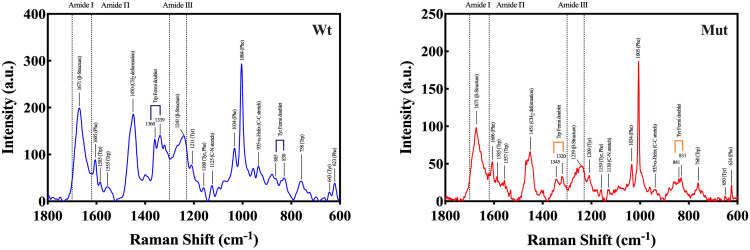
Fluorescence analysis of wild-type and p.R163C αB-crystallin proteins. Intrinsic fluorescence spectra of Tyr and Trp residues in wild-type and p.R163C mutant αB-crystallin at 27 °C, 37 °C, and 47 °C were collected. Excitation wavelengths were set to 280 nm for Tyr and 295 nm for Trp, with synchronous scanning intervals of 15 nm (Tyr) and 60 nm (Trp), and emission slit widths of 5/10 nm. 3D fluorescence and excitation-emission contour maps were utilized to further probe structural differences induced by the p.R163C mutation (right panel). Excitation-emission contour map of wild-type and p.R163C αB-crystallin, with excitation wavelengths ranging from 200 to 350 nm (5 nm increments) and emission wavelengths from 300 to 500 nm (1 nm increments), acquired at a scanning speed of 2400 nm/min and detector voltage of 800 V. Protein concentration for all fluorescence spectra was maintained at 0.15 mg/mL in buffer A.

The intrinsic fluorescence emission ability of proteins, due to the presence of aromatic amino acids provides valuable information about the structure and conformational changes of proteins. As shown in Fig 4, at temperatures of 27, 37, and 47 °C, the fluorescence spectra of Trp and Tyr of the p.R163C mutant protein and the wild-type protein do not differ significantly. With increasing temperature, the fluorescence emission intensity of both the wild-type and mutant proteins decreases due to the change in the overall protein structure. As the temperature rises, the protein structure changes, exposing aromatic amino acids to the aqueous environment, and leading to a decrease in fluorescence emission intensity. However, this reduction in emission intensity is not the same for both samples; in the wild-type protein, the fluorescence emission intensity decreases to a greater extent. The synchronous spectrum also shows a similar trend in the change in the environment around the Trp and Tyr amino acids of the wild-type and mutant proteins, indicating structural changes in these protein samples. A decreasing trend in fluorescence emission in the synchronous spectra is observed with increasing temperature. Therefore, the results suggest that the p.R163C mutation did not significantly alter the environment surrounding the aromatic residues in the protein structure.

The contour map reveals four prominent peaks. Peak a corresponds to first-order Rayleigh scattering (λ_ex_ = λ_em_), resulting from elastic scattering by solute and solvent molecules. Peak b represents second-order scattering (2λ_ex_ = λ_em_), which arises from light scattering by bulk particles in the sample. Peaks 1 and 2, observed in both the contour map and 3D protein spectra, primarily reflect the spectral characteristics of Trp and Tyr residues. These two peaks for Trp and Tyr residues were recorded at excitation/emission wavelengths (λ_ex_/ λ_em_) of 280/350 nm and 230/350 nm, respectively [49]. As shown in the contour map and 3D graph in Fig 4, the spectral pattern and fluorescence emission intensity of αB-crystallin remain largely unchanged due to the p.R163C mutation, with only a negligible decrease in the fluorescence emission intensity of the mutant protein observed compared to the wild type.

The ANS fluorescent probe was used to evaluate the hydrophobic surfaces exposed in the αB-crystallin protein due to the p.R163C mutation. This marker binds to hydrophobic regions of the protein, leading to an increase in fluorescence emission. As shown in S1 Fig, the hydrophobic surface exposure of the protein decreases as the temperature increases. Notably, the reduction in fluorescence intensity with increasing temperature, consistent with changes in intrinsic fluorescence, is more pronounced in the wild-type protein compared to the mutant. Overall, the fluorescence emission of the p.R163C mutant protein was higher than that of the wild-type protein, suggesting that the mutation reduces hydrophobic surface exposure in αB-crystallin. Unlike the wild-type protein, the mutant exhibited no significant change in hydrophobicity under increasing temperature or thermal stress conditions.

**Fig 5:**
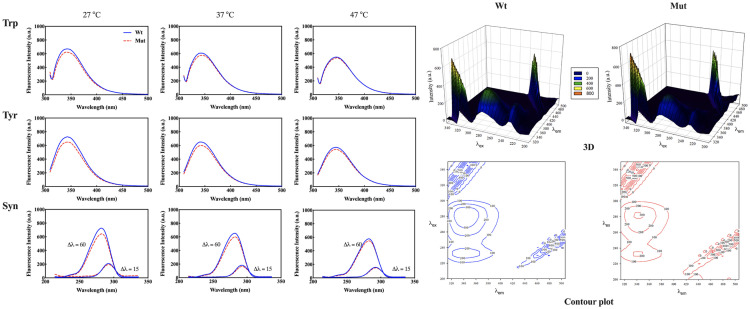
Structural dynamics of wild-type and p.R163C mutant αB-crystallin dimers: insights from molecular simulations. MD simulations were conducted using the CHARMM36 force field and HTMD/ACEMD platforms to analyze the dimeric structures of wild-type and p.R163C mutant αB-crystallin over 3000 ns, following a 4 ns equilibration phase. Systems were prepared at pH 7.4 with pdb2pqr and propka for protonation and charge assignment, then solved and neutralized in water using VMD tools. **(A)** Snapshot from the MD simulation of the p.R163C mutant dimer, illustrating the spatial arrangement of monomer units. The distance between the cysteine residues at position 163 (C163–C163) averages 73.18 ± 10.42 Å, precluding disulfide bond formation. **(B)** The inter-monomer angle in the p.R163C dimer is calculated as 157.12 ± 12.84°, reflecting slightly altered dimer geometry due to the mutation. **(C)** Additionally, the distance between the cysteine residues in the wild-type dimer stabilizes at ~25 Å over a 600 ns segment, with no evidence of intramolecular bonding, highlighting structural stability and subunit spacing differences induced by the p.R163C mutation.

MD simulations were also performed to investigate structural changes in the dimeric form of the p.R163C mutant of αB-crystallin. Based on the root-mean-square fluctuation (RMSF) analysis, the NTD of one monomeric subunit had lower values, suggesting a decrease in atomic oscillation and dynamic motion within this region (S2 Fig). A comparison of the average distance between the two residues at position 163 in the dimer form of wild-type and mutant αB-crystallin proteins shows a slight increase in the distance between the two monomeric subunits in the mutant protein compared to the wild-type. This distance was 68.46 ± 12.72 Å in the wild-type protein and 73.18 ± 10.42 Å in the p.R163C mutant protein (Fig 5A). In addition, the average angle between monomeric units in the dimeric conformation of wild-type and mutant αB-crystallin proteins was compared.

As shown in Fig 5B, the average angle between the vectors passing through the α carbon of glycine 95 and glutamate 117 residues in each protein subunit in the dimer of normal and p.R163C mutant proteins is 154.79 ± 11.43 and 157.12 ± 12.84 degrees, respectively, indicating a marginal increase in the angle between monomeric units in the dimer form of mutant αB-crystallin proteins. Next, the propensity of the monomers to form disulfide bonds in the dimer form of the mutant αB-crystallin protein was studied. To this end, through MD simulations with adaptive sampling, the distance between two cysteine residues was reduced in a controlled manner to investigate whether the cysteine residues in the p.R163C mutant could come into close enough proximity to form a disulfide bond. The results indicate that the two cysteine residues could come into proximity of 25 angstroms without any intramolecular disulfide bond being apparent between these two residues (Fig 5C).

### 3.3 Increased size of protein oligomers due to the p.R163C mutation

**Fig 6:**
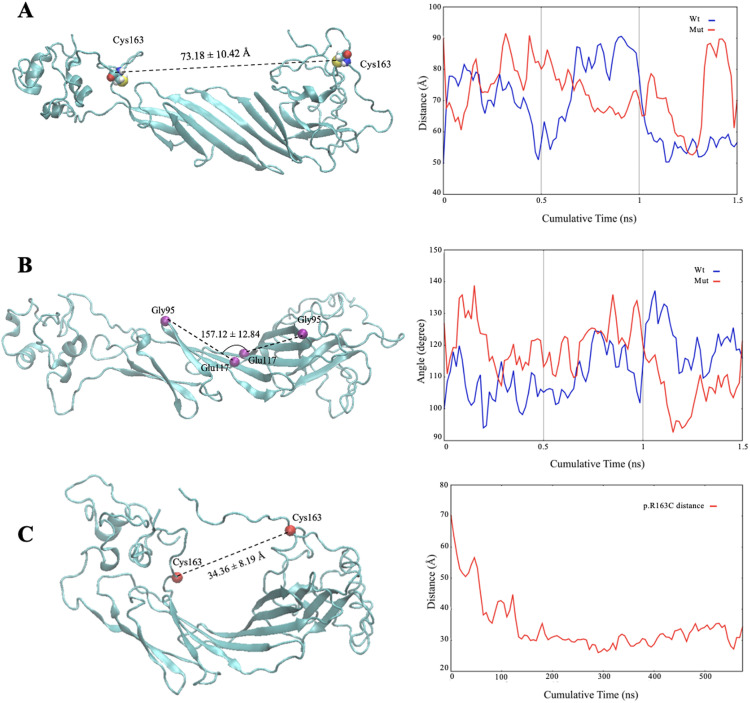
Investigation of oligomeric size distribution in the proteins. **(A)** DLS analysis of wild-type and p.R163C mutant αB-crystallin at 37 °C, performed on samples prepared at 1 mg/mL in buffer A. Scattering data were collected using a 532 nm laser at a 173° detection angle. Main plots show the volume-weighted size distribution of oligomers, revealing larger oligomeric species in the p.R163C mutant compared to the wild-type. Inset plots display the intensity-weighted scattering profiles, correlating light scattering intensity with oligomer size. **(B)** AFM imaging of quaternary structure and oligomeric size distribution for wild-type and p.R163C αB-crystallin. Protein samples, deposited onto mica discs, were imaged in non-contact mode using an ARA-AFM system. Particle size and morphology were quantified with Imager (v1.01) and Gwyddion (v2.62) software, providing complementary insights into the mutation’s impact on oligomerization.

Human αB-crystallin, as a dynamic protein, is able to exchange subunits and generate oligomeric structures [50]. DLS was used to evaluate the size of the oligomers formed in the protein samples. The effect of the p.R163C mutation on the formation of αB-crystallin protein oligomers at 37 °C is indicated in Fig 6A. As can be observed, the mutant can form oligomers with a larger size than the wild-type, with the hydrodynamic diameter of the wild-type and mutant being 17.7 nm and 22.8 nm, respectively. Moreover, AFM can measure the size of protein oligomers on a smooth, polished surface with angstrom accuracy and provide topographic images.

AFM images of wild-type and mutant oligomers (Fig 6B) are in accordance with the findings of DLS, with the average heights of wild-type and p.R163C proteins being 13.12 nm and 20.31 nm, respectively. These results suggest that substituting the arginine at position 163 with cysteine in human αB-crystallin increases the average oligomer size of this mutant protein relative to the wild-type protein.

### 3.4 Evaluation of the stability of p.R163C mutant αB-crystallin

**Fig 7:**
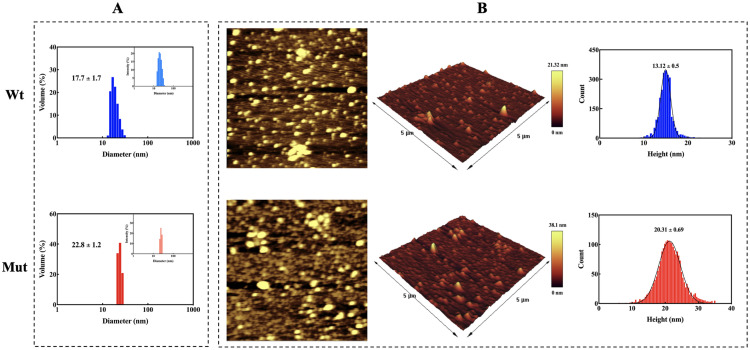
Chemical, thermal, and proteolytic stability of αB-crystallin proteins. **(A)** Chemical stability of wild-type and p.R163C αB-crystallin assessed by urea-induced denaturation. Protein samples (0.15 mg/mL in buffer A: 50 mM phosphate, pH 7.4) were incubated with 0–8 M urea for 18 hours at room temperature. Trp fluorescence emission (300–500 nm, excitation at 295 nm) was recorded, and the intensity ratio of unfolded to native states (I_U_/I_N_) was plotted against urea concentration. Data were fitted to a three-state denaturation model to derive free energy changes and overall stability (*ΔG°*). **(B)** Thermal stability of wild-type and p.R163C αB-crystallin evaluated by differential scanning calorimetry (DSC). Samples (1.5 mg/mL in buffer A) were heated from 25 to 85 °C at 1 °C/min, and changes in heat capacity (*ΔCp*) were plotted against temperature. Thermodynamic parameters (*ΔHº*, *ΔCp*, *ΔSº*, *T*_*m*_, *ΔGº*) were analyzed using CpCalc 2.1 software. **(C)** Proteolytic stability of wild-type and p.R163C αB-crystallin against α-chymotrypsin digestion. Proteins (1 mg/mL in buffer A) were incubated with α-chymotrypsin (100:1 w/w ratio) at 37 °C for 5, 10, 15, and 20 minutes, with reactions terminated by heating at 100 °C for 10 minutes. Samples (12.5 μg) were analyzed by 12% SDS-PAGE, stained, and destained to visualize degradation patterns.

The chemical stability of wild-type and mutant p.R163C proteins was studied by measuring the fluorescence emission of Trp at different urea concentrations and analyzing the denaturation of the proteins. To obtain stability parameters, the ratio of the maximum emission wavelength (λ_max_) of the protein in the fully unfolded to the fully native state (*I*_*U*_*/I*_*N*_) was plotted against different urea concentrations (Fig 7A).

Then, the various parameters were extracted using formula 1. The quantitative results related to the transition point from the native to the denatured state (C_1/2_) and *ΔG^°^* are shown in Table 2. C_1/2_ of the p.R163C mutant protein shows a lower urea concentration than the wild-type protein, which may indicate a lower stability of the mutant protein against chemical stress. Additionally, the comparison of *ΔG^°^* values shows that there is not much difference between the chemical stability of the mutant and wild-type forms of this protein. To measure the thermal stability of the p.R163C mutant protein and compare it with the natural protein, the DSC method was performed. Given that in the oligomerization process, protein subunits can cooperatively interact with each other and form a protein complex, a single peak in the DSC plot suggests the separation of protein subunits and their simultaneous denaturation. However, the presence of two peaks most likely indicates the separation of protein subunits in the first stage and then their denaturation in the second stage [51,52] (Fig 7B). The p.R163C mutant protein exhibits reduced thermal stability compared to the wild-type, with a lower melting temperature (*T*_m_) of 60.7 °C, in contrast to 63.4 °C for the wild-type. Additionally, the mutant protein shows a significant decrease in the standard enthalpy change (Δ*H*^°^), further indicating compromised structural stability. The thermodynamic parameters of thermal stability are summarized in Table 3.

**Table 2:** *ΔG°* and C_1/2_ values of various αB-crystallin samples derived from equilibrium urea unfolding analysis.

αB-crystallin	*ΔG°* (kcal/mol)	C_1/2_ (M)
Wild-type	6.89 ± 0.24	3.19 ± 0.16
p.R163C	6.32 ± 0.31	2.89 ± 0.12

**Table 3:** Thermodynamic parameters of αB-crystallin protein samples determined through DSC analysis.

αB-crystallin	*ΔG°* (kcal/mol)	*ΔH* (kcal/mol)	*ΔS* (kcal/K.mol)	*T*_*m1*_ (°C)	*T*_*m2*_ (°C)
Wild-type	3.804	34.2	0.102	63.4	–
p.R163C	**1.**652	24.3	0.076	60.7	47.7

An enzymatic digestion method with α-chymotrypsin protease was used to assess the proteolytic stability of the p.R163C mutant protein and compare it with the wild-type protein. Both protein samples were exposed to the enzyme for 5, 10, 15, and 20 minutes, and their digestion patterns were analyzed via SDS-PAGE. As shown in Fig 7C, the mutant protein exhibits greater resistance to proteolysis, with higher band intensity at 15 and 20 minutes. Enzymatic cleavage generates smaller fragments that bind with αB-crystallin oligomers, forming higher molecular weight species. The p.R163C mutation likely alters the protein’s structure, reducing the exposure of cleavage sites and enhancing resistance to enzymatic digestion.

### 3.5 p.R163C mutation causes the augmentation of amyloidogenic properties in human αB-crystallin

**Fig 8:**
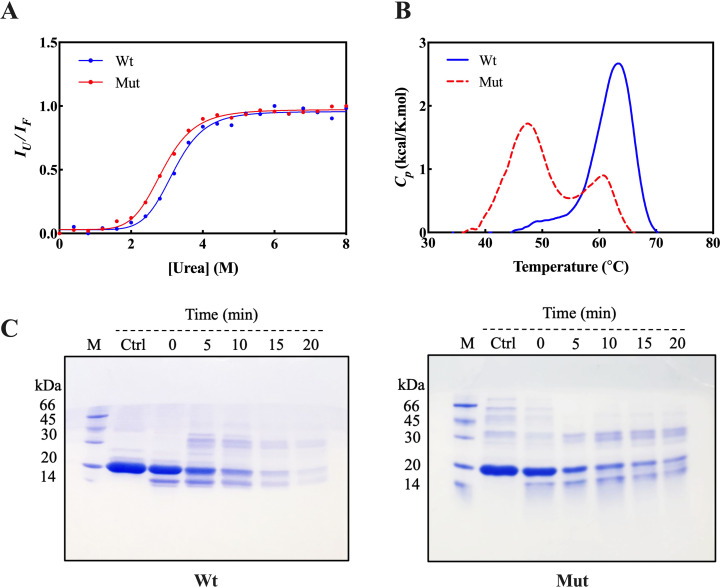
Comparative analysis of amyloid fibril formation of the proteins. Wild-type and p.R163C αB-crystallin proteins (2 mg/mL) were subjected to thermochemical stress (60 °C, 1 M guanidine hydrochloride, 4 days) to induce amyloid fibril formation. Fibril samples were centrifuged (14,000 rpm, 30 min, 4 °C), resuspended in buffer A (50 mM phosphate, pH 7.4), and deposited onto mica discs for AFM analysis in non-contact mode using an ARA-AFM system. **(A)** Comparison of the amount of amyloid fiber formation in wild-type and mutant p.R163C αB-crystallin protein before and after thermochemical stress using fluorescence microscopy. **(B)** AFM images reveal the morphology, length, and diameter of amyloid fibrils formed by wild-type and p.R163C αB-crystallin. Furthermore, the particle size distribution of amyloid fibrils derived from AFM data, quantified using Imager (v1.01) and Gwyddion (v2.62) software, comparing fibril dimensions between wild-type and mutant proteins.

ThT binds to β-sheet-rich fibrils, increases fluorescence emission, and allows the detection of amyloid formation. Thermal (60 °C) and chemical stress (1 M guanidine hydrochloride) led to increased fluorescence, indicating enhanced fibril formation. The mutant protein exhibited higher fluorescence overall, suggesting greater amyloid propensity or an increase in β-sheet content, although the differences were not statistically significant (S3 Fig). To further clarify, while the p.R163C mutant protein exhibited higher ThT fluorescence intensity compared to the wild-type, indicating a trend toward increased amyloid fibril formation, these differences were not statistically significant (p > 0.05, S3 Fig). However, fluorescence microscopy and AFM analyses (Fig 8A and 8B) provide compelling evidence of enhanced amyloidogenicity, revealing that the mutant protein forms longer, thicker, and denser fibrils under thermochemical stress conditions, which is consistent with its elevated β-sheet content and reduced structural stability. These findings align with secondary structure analyses, which show a slight increase in the p.R163C mutant. Fluorescence microscopy of ThT-stained samples before and after stress application revealed amyloid fibril formation in both wild-type and mutant proteins. Before stress, only a few protein plaques were observed. Following stress application, the number and distribution of fibrils increased, with the p.R163C mutant showing a slightly higher but not remarkable fibril presence compared to the wild-type protein (Fig 8A).

Besides, morphological evaluation of amyloid fibril protein samples was examined using atomic force microscopy. The p.R163C mutant αB-crystallin protein forms dense and intertwined amyloid fibrils under thermochemical stress conditions, which, in terms of appearance, the fibrils are somewhat longer and thicker than those formed in the wild-type (Fig 8B).

### 3.6 Enhanced chaperone activity and cellular protection by the p.R163C mutant protein

**Fig 9:**
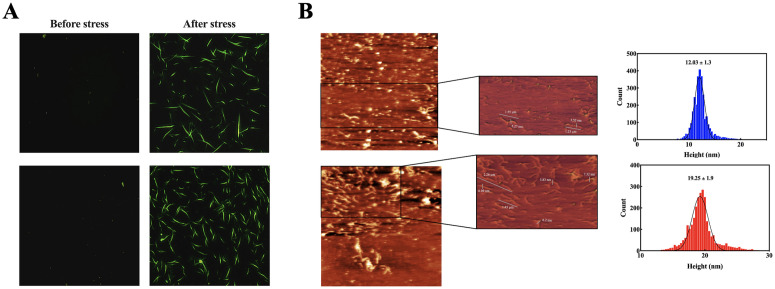
Chaperone activity and cytoprotective effects of the αB-crystallin proteins. **(A)** Chaperone activity of wild-type and p.R163C αB-crystallin assessed by monitoring aggregation prevention of target proteins: catalase (0.3 mg/mL, 60 °C), γ-crystallin (0.25 mg/mL, 60 °C), and insulin (0.3 mg/mL, 40 °C with 20 mM DTT). Aggregation was quantified by light absorbance at 360 nm using a Cary 100 Bio UV-Vis spectrophotometer, with protection efficiency calculated as the percentage reduction in the area under the curve. The p.R163C mutant exhibited enhanced anti-aggregation activity compared to wild-type. **(B)** Preservation of α-glucosidase enzymatic activity by wild-type and p.R163C αB-crystallin. α-Glucosidase (0.2 U/mL) was incubated at 46 °C with or without 0.05 mg/mL αB-crystallin, and activity was measured every 5 minutes for 30 minutes, demonstrating differential stabilization by the mutant. **(C)** Cytoprotective role of αB-crystallin against H₂O₂-induced oxidative stress in SH-SY5Y neuroblastoma cells. Cells were pre-treated with 12.5 or 25 μM αB-crystallin (wild-type or p.R163C) for 2 hours in serum-free DMEM/F12, followed by 18-hour exposure to 0.8 mM H_2_O_2_. Cell viability was assessed via MTT assay, measuring formazan absorbance at 570 nm after dissolution in DMSO, revealing enhanced protection by the p.R163C mutant protein.

The chaperone activity of the p.R163C mutant was assessed *in vitro* by monitoring the aggregation of target proteins (catalase, insulin, and γ-crystallin) under chemical and thermal stress. αB-crystallin prevents aggregation by binding target proteins, which was quantified using UV-Vis spectroscopy at 360 nm. It is shown in Fig 9A that the p.R163C mutation remarkably enhanced chaperone activity, likely due to structural changes that improve protein-protein interactions. *In vivo*, the mutant’s chaperone function was evaluated by comparing bacterial survival under heat shock (50 °C vs. 37 °C). Bacteria expressing the mutant protein exhibited greater heat resistance than those with the wild-type or empty vector, indicating superior chaperone activity (S4A Fig). The mutant also enhanced the stability of α-glucosidase, an enzyme with a half-life of 15 minutes at 46 °C. Compared to the wild-type, p.R163C provided stronger protection against thermal stress, preserving enzyme function more effectively (Fig 9B). Long-term incubation (10 days, 37 °C) of αB-crystallin with γ-crystallin revealed that while both wild-type and mutant proteins reduced aggregation, the mutant retained more γ-crystallin in the soluble fraction, confirming its superior chaperone function (S4B Fig).

Additionally, the cellular protection activity of the mutant was studied via MTT assay in SH-SY5Y cells exposed to H_2_O_2_ (0.8 mM, 18h). Both wild-type and mutant proteins improved cell survival, but the mutant offered greater protection. These findings, as can be seen in Fig 9C, suggest a direct link between enhanced chaperone activity and increased protective function.

## 4. Discussion

Several mutations in the αB-crystallin encoding gene (*CRYAB*) have been linked to different types of myopathies, including DCM. A recent discovery has identified a novel point mutation in *CRYAB*, designated as R163C, located within the palindromic peptide sequence of the protein’s CTD, which has been implicated in the development of DCM [21,22]. This mutation is crucial for the electrostatic interactions between αB-crystallin and its target proteins. Additionally, the domain where this mutation occurs is significant for protein oligomerization due to its high flexibility [13,53]. One of the main sequences that plays a vital role in the chaperone activity of human αB-crystallin and is known as the mini-chaperone is the palindromic sequence 156ERTIPITRE164. Previous findings confirmed that a part of this peptide sequence, arginine number 157 to arginine number 163, is stuck to the β4/β8 groove of the αB-crystallin protein in an oligomeric state, which is one of the major substrate-binding regions and has a direct effect in protein-protein interactions [18]. Since this protein naturally lacks cysteine, it is anticipated that the substitution of the conserved arginine at position 163 with cysteine, along with the formation of disulfide bonds at that site in the human αB-crystallin protein, will lead to structural and functional alterations. The use of various spectroscopic methods, such as CD, FTIR, and Raman, clearly showed that the secondary structure content of the mutant protein does not change significantly compared to the wild-type protein counterpart (Fig 2). This protein has a dynamic structure, and subunit exchange occurs regularly using dimeric and hexameric building blocks to create the oligomeric form of the αB-crystallin protein. MD studies revealed that the angle formed between the two mutant αB-crystallin monomers in the dimer state is similar to that of the wild-type and is approximately 157 degrees (Fig 5). Also, the distance between these two subunits is equivalent to the wild-type protein and is 73.18 Å. Nonetheless, DLS and AFM studies showed that the p.R163C mutation leads to the formation of larger oligomers with a higher number of subunits than the wild-type human αB-crystallin protein (Fig 6). Therefore, the appearance of the cysteine at position 163 in the human αB-crystallin protein may give rise to new features and, consequently, the ability of this protein to form larger oligomeric structures. MD analyses also suggested that in the p.R163C mutant dimer, monomeric units can be 25 Å apart without any intramolecular disulfide bonds being formed between these two units (Fig 5). A notable observation in our study is the apparent discrepancy between the MD simulations and the SDS-PAGE results concerning disulfide bond formation in the p.R163C mutant. MD simulations indicate that the Cys163 residues in the mutant dimer are separated by approximately 25 Å (Fig 5C), which is too distant for intramolecular disulfide bond formation, as disulfide bonds typically require a distance of 3–7.5 Å [54]. In contrast, SDS-PAGE under non-reducing conditions reveals a higher molecular weight band that is consistent with the presence of disulfide-linked dimers or oligomers (Fig 1C). This discrepancy can be reconciled by considering that the higher molecular weight band likely results from inter-dimer disulfide linkages, where Cys163 residues from different dimers within the larger oligomeric assemblies of the mutant protein come into close proximity. The p.R163C mutant’s tendency to form larger oligomers, as evidenced by DLS and AFM analyses (Fig 6A and 6B), supports this interpretation, as the flexible C-terminal domain containing Cys163 may adopt conformations that facilitate such interactions in the oligomeric state. The MD simulations, which focus on the dimeric state, do not account for the dynamic interactions between dimers in these larger assemblies, explaining the absence of predicted disulfide bonds.

Investigation of the hydrophobic surfaces of the protein samples using the ANS probe showed that at 27 °C, despite the larger size of the p.R163C mutant protein, its accessible hydrophobic surfaces are less than those of the wild-type, and these regions are probably buried within the mutant protein. With increasing temperature, the hydrophobic surfaces in the wild-type protein decreased significantly, while no significant change was observed in the mutant protein (S1 Fig). Given that most interactions between chaperone protein aggregates are mediated by hydrophobic interactions, this could lead to increased chaperone activity of the mutant protein under thermal stress conditions. Comparison of the intrinsic fluorescence spectra of wild-type and mutant αB-crystallin proteins also showed that with increasing temperature to a certain extent, the three-dimensional structure of the mutant protein changes less and is more stable to this change (Fig 4). The results of the thermal stability study of the p.R163C mutant protein by the DSC method revealed that the separation of the mutant αB-crystallin protein subunits occurs at a different temperature from the denaturation of the monomeric state of the protein. This is while these two events occur at a certain temperature in the wild-type protein (Fig 7B). As the temperature increases, the subunits dissociate and convert to the monomeric state, making the internal hydrophobic parts of the protein accessible. Due to the major effect of the urea denaturant on hydrogen bonds and hydrophobic interactions, the substitution of arginine with cysteine reduced the chemical stability of the mutant αB-crystallin protein (Fig 7A). Besides, the conformation of the p.R163C mutant protein has changed in such a way that fewer cleavage sites for the protease chymotrypsin are available to this enzyme, and the target protein shows greater stability against enzymatic digestion. The thermal and chemical instability of the p.R163C mutant αB-crystallin may promote amyloid fibril formation under stress (Fig 7).

The increased chaperone activity of the p.R163C mutant αB-crystallin (Fig 9A) may not be entirely beneficial, as excessive binding to target proteins can form abnormal aggregation. The binding strength between the chaperone and the target protein is considered optimal within a specific intermediate range [55]. Studies have demonstrated that an excessive increase in binding affinity, which is often associated with heightened chaperone activity *in vitro*, can lead to unforeseen outcomes *in* *vivo*, such as coaggregation of the chaperone and the target protein [55–57]. Our findings indicate that the mutant protein enhances cell survival compared to the wild-type protein (Fig 9C). This effect may be attributed to its stronger interaction with specific proteins involved in apoptosis, which have previously been identified as target proteins of αB-crystallins. Prior research has indicated a link between elevated chaperone activity and the capacity of chaperones to help cancer cells survive under stress [58,59]. As a result, the heightened chaperone activity of this protein due to the mutation—given its contribution to cancer cell survival and its ubiquitous presence across different tissues—could pose serious implications for its role in cardiomyopathy. The increased chaperone activity of the p.R163C mutant, as demonstrated by its enhanced ability to inhibit aggregation of catalase, insulin, and γ-crystallin (Fig 9A), protect α-glucosidase from thermal denaturation (Fig 9B), and improve cell survival under oxidative stress (Fig 9C), is likely driven by its larger oligomeric state and structural changes (Fig 6, Table 1). However, this heightened chaperone efficiency may pose pathophysiological risks, particularly in the context of DCM. Overly tight binding to client proteins, as observed with hyperactive chaperones, can lead to excessive sequestration, disrupting the normal function of critical proteins, or result in the formation of cytotoxic coaggregates [55–57]. In cardiac muscle cells, the p.R163C mutant’s enhanced interaction with desmin, a key sarcomeric protein, could impair filament assembly or promote pathological aggregation, contributing to sarcomere disorganization and contractile dysfunction characteristic of DCM [60]. The context-dependent nature of these outcomes depends on factors such as cellular stress, the specific client proteins involved, and the tissue environment. For example, while the mutant’s chaperone activity enhances cell survival in SH-SY5Y cells under oxidative stress (Fig 9C), in cardiac tissue under chronic stress, it may exacerbate protein misfolding or aggregation, highlighting the need for further studies in cardiac-relevant systems to elucidate these risks [24,26].

While our study provides detailed insights into the structural and functional consequences of the p.R163C mutation in αB-crystallin, a limitation is the absence of experiments in cardiac-relevant systems. Such systems would enable direct validation of the mutation’s impact on cardiac muscle function and its role in DCM pathogenesis. Future studies using cardiac-specific models are essential to confirm the mechanistic link between the observed molecular changes and cardiomyopathy. Additionally, the mutation’s effect on interactions with cardiac-specific proteins, particularly desmin, warrants further exploration. αB-crystallin is critical for stabilizing desmin filaments, which are essential for maintaining sarcomere integrity in cardiac muscle [24,60]. The substitution of arginine 163 with cysteine may disrupt this interaction, potentially leading to desmin filament misassembly or aggregation, which could impair sarcomere organization and contribute to the dilated phenotype observed in DCM [23]. Furthermore, the enhanced chaperone activity of the p.R163C mutant, while beneficial in preventing protein aggregation *in vitro*, may lead to excessive binding to desmin or other sarcomeric proteins, such as actin or titin, potentially causing coaggregation and exacerbating cardiac dysfunction [55–57]. Prior studies using cell-based models have shown that αB-crystallin mutations alter subcellular localization and disrupt interactions with cardiac proteins, contributing to cardiomyopathy [17,26]. Similarly, animal models have demonstrated the role of αB-crystallin in maintaining cardiac muscle homeostasis under stress, with mutations leading to contractile dysfunction [24]. These findings emphasize the need for cardiac-specific investigations to fully elucidate the pathogenic mechanisms of the p.R163C mutation.

**Scheme 2:**
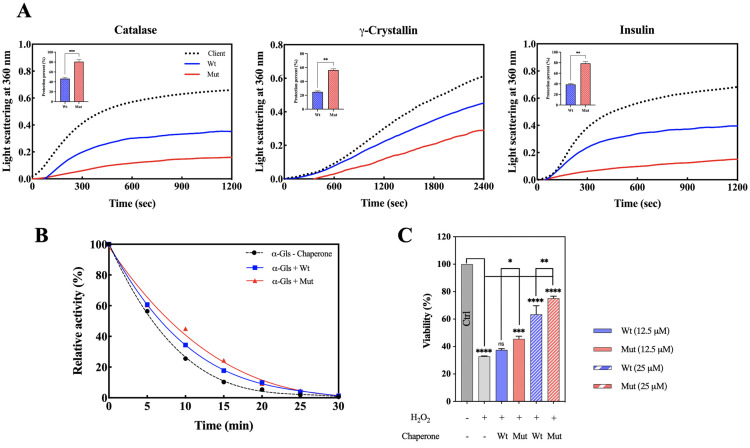
Altered structure and function of human αB-crystallin due to the p.R163C mutation. The p.R163C mutation is linked to DCM, characterized by an enlarged left ventricle and compromised cardiac muscle function. This mutant protein tends to form larger oligomers than its wild-type counterpart. Although it exhibits decreased chemical and thermal stability, it demonstrates greater proteolytic stability and shows an increased tendency for fibrillation under thermochemical stress conditions. Furthermore, the p.R163C mutant αB-crystallin protein demonstrates an enhanced ability to inhibit the aggregation of target proteins.

In summary, the pathogenic mutation p.R163C compromises the chemical and thermal stability of the human αB-crystallin protein, facilitates the formation of larger oligomers, and amplifies its chaperone activity (Scheme 2).

Additionally, the conserved arginine at position 163 plays a crucial role in the interaction between αB-crystallin and desmin [60]. Mutating this residue may disrupt the interaction between the altered αB-crystallin and desmin in cardiac muscle cells. Alongside the previously mentioned structural and functional changes, this disruption provides insight into the pathogenic mechanisms of p.R163C in the development of cardiomyopathy.

## 5. Conclusion

Overall, the findings of this study suggest that the conserved arginine 163 in the palindromic sequence of the C-terminus of human αB-crystallin is crucial for the protein’s structure and function. Substituting this arginine with cysteine disrupts the oligomerization pattern, reduces thermal and chemical stability, and enhances the chaperone activity of αB-crystallin. The increased chaperone activity of the mutant protein, while potentially protective against protein aggregation and cellular stress, may also lead to excessive sequestration of client proteins or coaggregation, which could be deleterious in certain contexts, such as cardiac muscle tissue where interactions with desmin are critical. These alterations in the mutant protein, combined with the established role of arginine 163 in its interaction with desmin, clarify its pathogenic mechanism in cardiomyopathy. However, considering that αB-crystallin interacts with multiple proteins involved in apoptosis, its highlighted ability to promote cancer cell survival raises significant concerns. Thus, it is essential to undertake a thorough investigation of mutant αB-crystallins that demonstrate increased chaperone activity and improved cell survival in order to fully comprehend their context-dependent effects in various disease states.

## Supporting information

S1 Fig Temperature-dependent hydrophobic surface exposure of the αB-crystallin proteins assessed by ANS fluorescence. Wild-type and p.R163C αB-crystallin (0.15 mg/mL in buffer A: 50 mM phosphate, pH 7.4) were incubated with 100 µM 8-anilino-1-naphthalenesulfonic acid (ANS) for 30 minutes in the dark. Fluorescence emission spectra (400–600 nm, excitation at 365 nm) were recorded using a Varian Cary Eclipse spectrofluorometer at 27 °C, 37 °C, and 47 °C, with slit widths of 5/10 nm. Plots illustrate ANS fluorescence intensity as a function of temperature, revealing a pronounced decrease in hydrophobic surface exposure in wild-type protein with increasing temperature, contrasted by minimal change in the p.R163C mutant. Higher baseline fluorescence in the mutant suggests reduced hydrophobic exposure compared to wild-type, consistent with intrinsic fluorescence trends.
(TIF)

S2 Fig Root-mean-square fluctuation (RMSF) analysis of wild-type and p.R163C mutant αB-crystallin dimers from molecular dynamics simulations. Molecular dynamics simulations of wild-type and p.R163C αB-crystallin dimers were performed using the CHARMM36 force field and HTMD/ACEMD over 3000 ns, following a 4 ns equilibration phase. Systems were solvated in water at pH 7.0, neutralized, and analyzed for atomic fluctuations. RMSF plots of the N-terminal domain across residues show reduced values in one monomeric subunit of the p.R163C mutant, indicating lower dynamic motion and structural rigidity compared to wild-type. Data highlights the mutation’s stabilizing effect on specific regions of the dimer.
(TIF)

S3 Fig Thioflavin-T (ThT) fluorescence analysis of amyloid fibril formation in wild-type and p.R163C mutant αB-crystallin under thermochemical stress. Wild-type and p.R163C αB-crystallin (2 mg/mL) were incubated at 60 °C with 1 M guanidine hydrochloride for 4 days to promote amyloid fibril formation. Samples (0.15 mg/mL) were mixed with 20 µM ThT and incubated in the dark for 5 minutes, after which fluorescence emission (450–600 nm, excitation at 440 nm) was recorded using a Varian Cary Eclipse spectrofluorometer. The plot shows increased ThT fluorescence in the p.R163C mutant compared to wild-type after stress, indicating a higher propensity for amyloid formation or increased β-sheet content, although the differences are not statistically significant. Pre-stress controls exhibited minimal fluorescence for both variants.
(TIF)

S4 Fig *In vivo* and long-term chaperone activity assessment of wild-type and p.R163C mutant αB-crystallin. **(A)** Heat shock resistance was assessed in *E. coli* expressing either wild-type or p.R163C αB-crystallin. Cells were grown at 37 °C (control) or subjected to heat stress at 50 °C following IPTG induction, and colony survival was measured relative to an empty vector control. The bar plots indicate that cells expressing the mutant exhibited enhanced survival, suggesting superior chaperone activity. Expression levels were confirmed by SDS-PAGE. **(B)** Long-term aggregation protection of γ-crystallin by αB-crystallin. Wild-type and p.R163C αB-crystallin (1 mg/mL) were co-incubated with γ-crystallin (1:1 ratio) at 37 °C for 10 days. Post-centrifugation (14,000 rpm, 10 min, 4 °C), soluble fractions were analyzed by SDS-PAGE, revealing greater retention of γ-crystallin in the supernatant with the p.R163C mutant, indicating enhanced chaperone function over wild-type.
(TIF)
