# Supplementary figures and images for: Structural and functional impact of the p.R163C mutation in the conserved palindromic motif within the C-terminal domain of human αB-crystallin
Source: PLoS One. 2025 Jul 14;20(7):e0326025. doi: 10.1371/journal.pone.0326025 (PMC12258569; doi:10.1371/journal.pone.0326025)

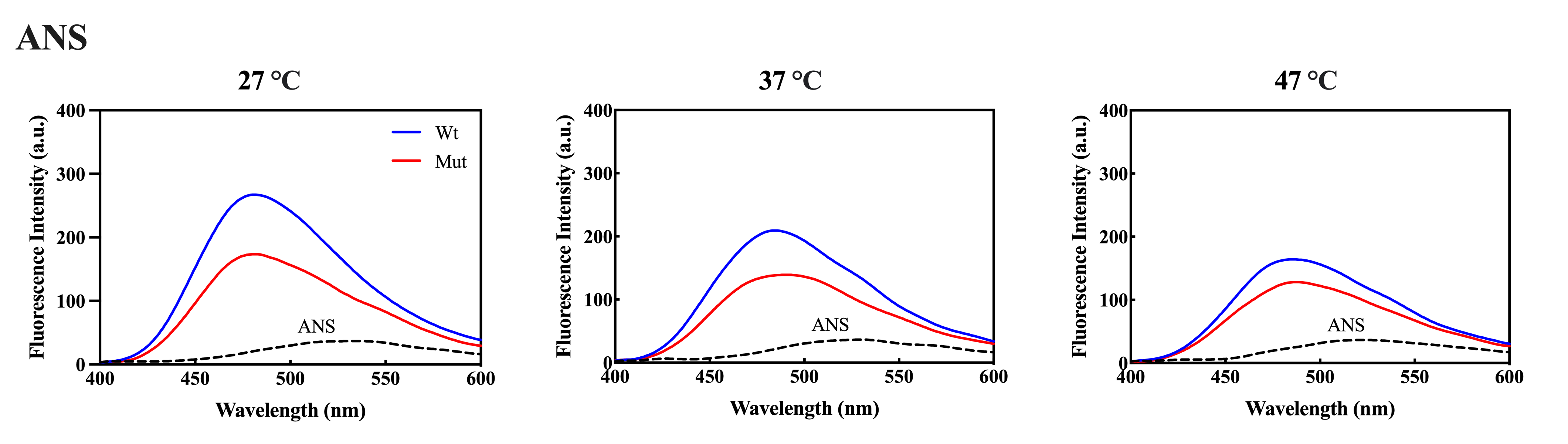

Supplement: S1 Fig — Wild-type and p.R163C αB-crystallin (0.15 mg/mL in buffer A: 50 mM phosphate, pH 7.4) were incubated with 100 µM 8-anilino-1-naphthalenesulfonic acid (ANS) for 30 minutes in the dark. Fluorescence emission spectra (400–600 nm, excitation at 365 nm) were recorded using a Varian Cary Eclipse spectrofluorometer at 27 °C, 37 °C, and 47 °C, with slit widths of 5/10 nm. Plots illustrate ANS fluorescence intensity as a function of temperature, revealing a pronounced decrease in hydrophobic surface exposure in wild-type protein with increasing temperature, contrasted by minimal change in the p.R163C mutant. Higher baseline fluorescence in the mutant suggests reduced hydrophobic exposure compared to wild-type, consistent with intrinsic fluorescence trends. (TIF) [file pone.0326025.s001.tif]

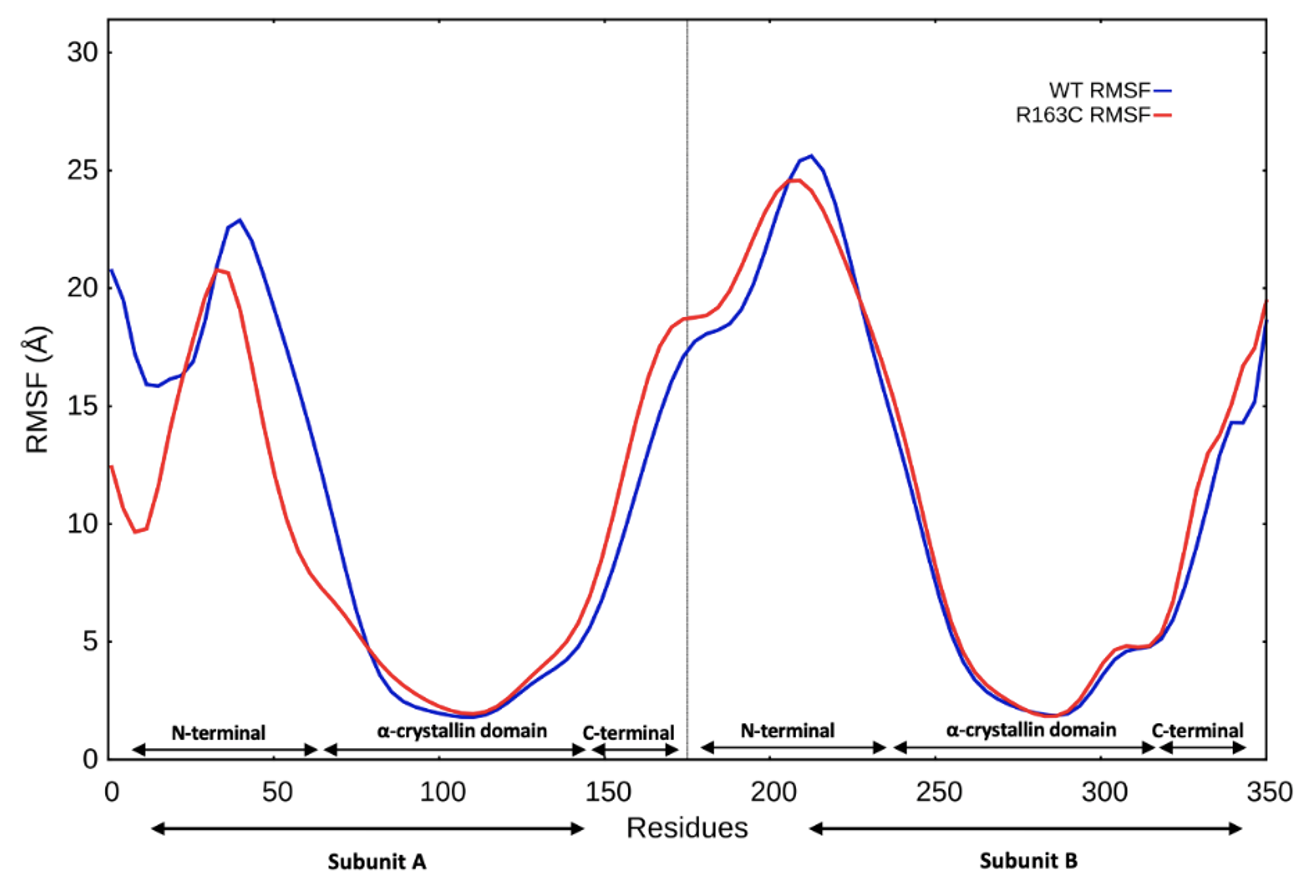

Supplement: S2 Fig — Molecular dynamics simulations of wild-type and p.R163C αB-crystallin dimers were performed using the CHARMM36 force field and HTMD/ACEMD over 3000 ns, following a 4 ns equilibration phase. Systems were solvated in water at pH 7.0, neutralized, and analyzed for atomic fluctuations. RMSF plots of the N-terminal domain across residues show reduced values in one monomeric subunit of the p.R163C mutant, indicating lower dynamic motion and structural rigidity compared to wild-type. Data highlights the mutation’s stabilizing effect on specific regions of the dimer. (TIF) [file pone.0326025.s002.tif]

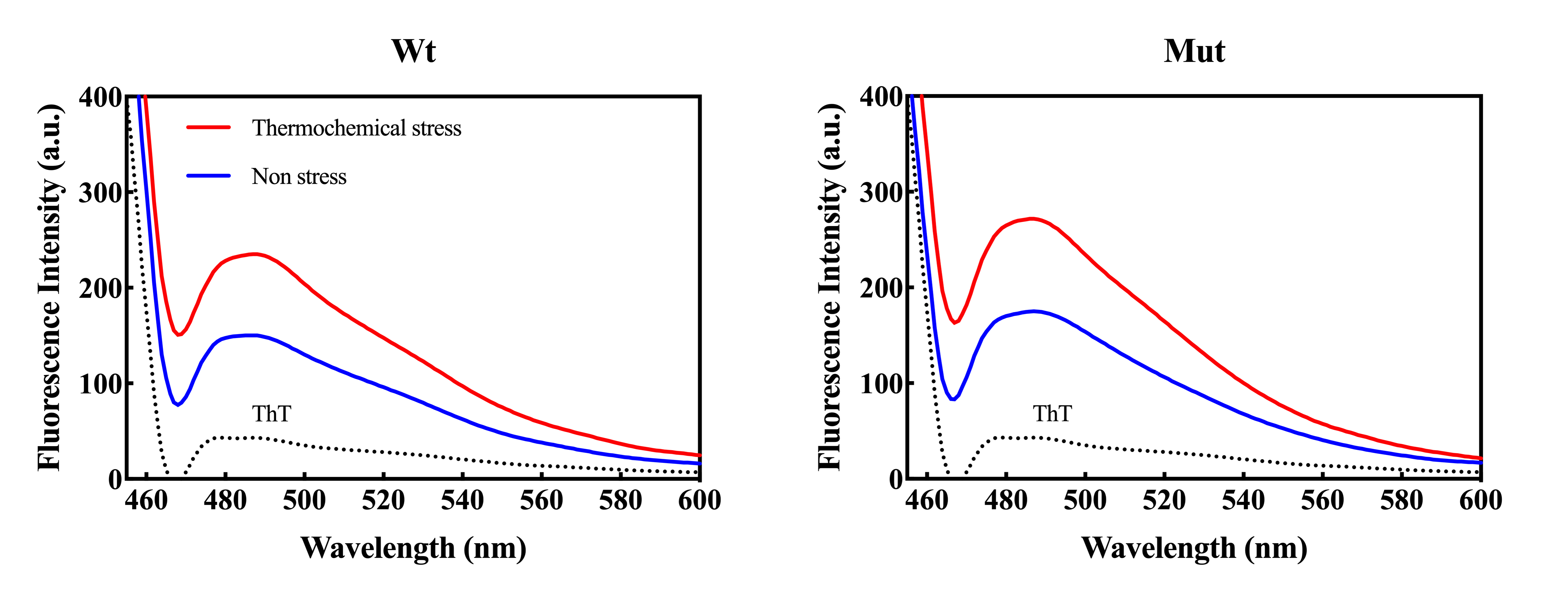

Supplement: S3 Fig — Wild-type and p.R163C αB-crystallin (2 mg/mL) were incubated at 60 °C with 1 M guanidine hydrochloride for 4 days to promote amyloid fibril formation. Samples (0.15 mg/mL) were mixed with 20 µM ThT and incubated in the dark for 5 minutes, after which fluorescence emission (450–600 nm, excitation at 440 nm) was recorded using a Varian Cary Eclipse spectrofluorometer. The plot shows increased ThT fluorescence in the p.R163C mutant compared to wild-type after stress, indicating a higher propensity for amyloid formation or increased β-sheet content, although the differences are not statistically significant. Pre-stress controls exhibited minimal fluorescence for both variants. (TIF) [file pone.0326025.s003.tif]

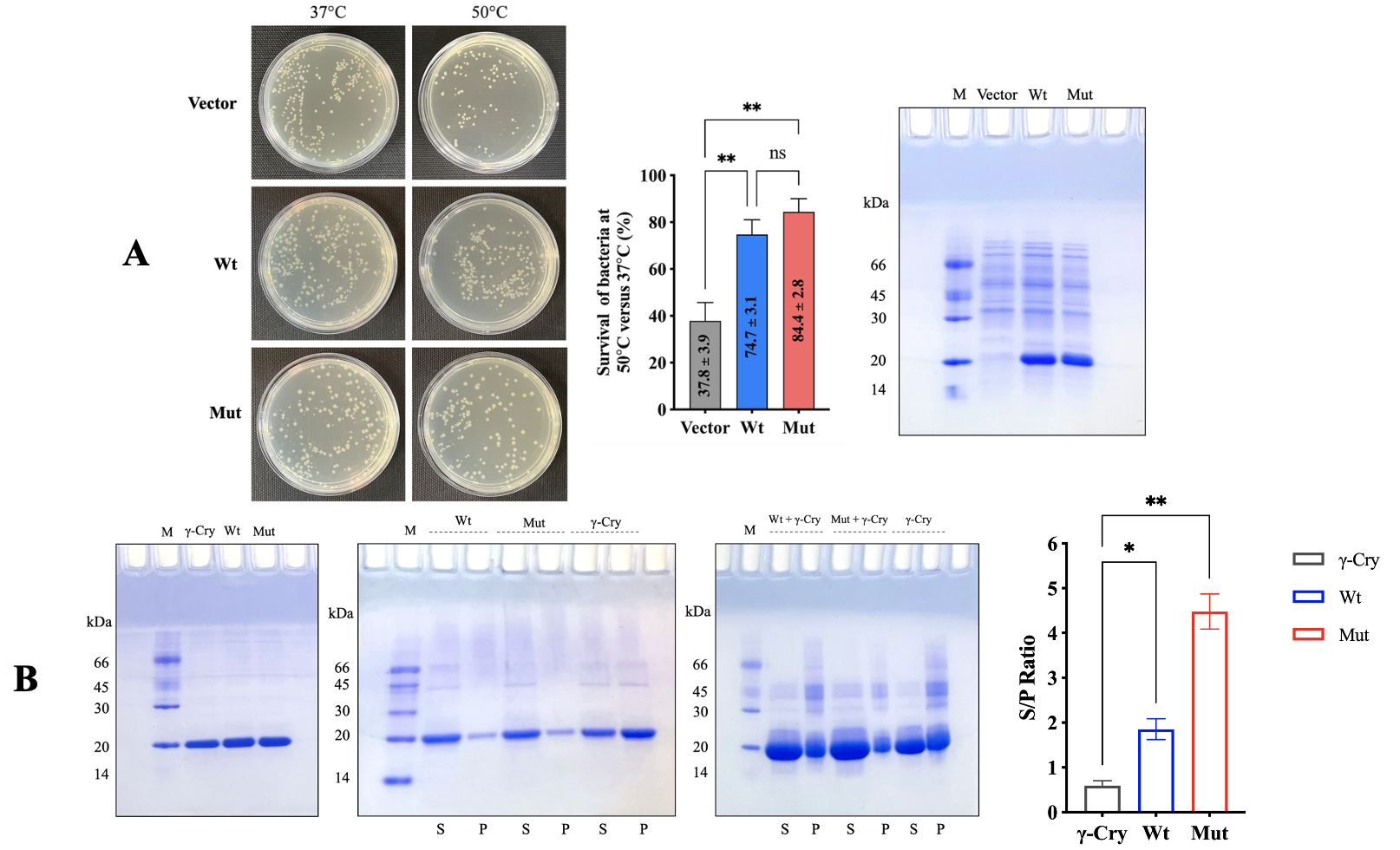

Supplement: S4 Fig — (A) Heat shock resistance was assessed in E. coli expressing either wild-type or p.R163C αB-crystallin. Cells were grown at 37 °C (control) or subjected to heat stress at 50 °C following IPTG induction, and colony survival was measured relative to an empty vector control. The bar plots indicate that cells expressing the mutant exhibited enhanced survival, suggesting superior chaperone activity. Expression levels were confirmed by SDS-PAGE. (B) Long-term aggregation protection of γ-crystallin by αB-crystallin. Wild-type and p.R163C αB-crystallin (1 mg/mL) were co-incubated with γ-crystallin (1:1 ratio) at 37 °C for 10 days. Post-centrifugation (14,000 rpm, 10 min, 4 °C), soluble fractions were analyzed by SDS-PAGE, revealing greater retention of γ-crystallin in the supernatant with the p.R163C mutant, indicating enhanced chaperone function over wild-type. (TIF) [file pone.0326025.s004.tif]
